# Is [F-18]-fluorodeoxyglucose FDG-PET/CT better than ct alone for the preoperative lymph node staging of muscle invasive bladder cancer?

**DOI:** 10.1590/S1677-5538.IBJU.2014.0579

**Published:** 2016

**Authors:** Mete Uttam, Nayak Pravin, Bhattacharya Anish, Kakkar Nandita, Mandal Arup

**Affiliations:** 1 Department of Urology, PGIMER, Chandigarh; 2 Department of Nuclear Medicine PGIMER, Chandigarh; 3 Department of Histopathology, PGIMER, Chandigarh

**Keywords:** Urinary Bladder Neoplasms, Positron-Emission Tomography, Tomography, X-Ray Computed, Carcinoma, Transitional Cell

## Abstract

**Objective:**

To evaluate whether the use of [F-18]-FDG-PET/CT can accurately predict pelvic lymph node metastasis in patients with muscle invasive TCC of the bladder undergoing radical cystectomy.

**Materials and Methods:**

Fifteen patients with muscle invasive bladder cancer had undergone FDG-PET/CT scan from the skull base to the mid-thighs after IV injection of 6.5MBq (Mega-Becquerel)/Kg of FDG. After intravenous hydration IV furosemide was given to overcome the difficulties posed by urinary excretion of 18F-FDG. PET/CT data were analyzed as PET and CT images studied separately as well as fused PET/CT images. The imaging findings were correlated with the histopathology of the nodes (gold standard).

**Results:**

CT and FDG-PET had demonstrated positive lymph nodes in 9 & 8 patients respectively. Among the 15 patients 3 had documented metastasis on histopathology. Both CT and PET could detect the nodes in all these 3 patients (100% sensitivity). Nodes were histologically negative amongst 6&5 patients who had node involvement by CT and PET respectively. Therefore, specificity, positive predictive value (PPV) & negative predictive value (NPV) for CT and PET/CT were 50%, 33.3%, 100% and 58.3%, 37.5%, 100% respectively.

**Conclusion:**

The theoretical advantage of this cutting edge technology for whole body imaging has not been translated into clinical practice as we found minimal advantage of combined FDG-PET/CT over CT alone for nodal staging of muscle invasive bladder cancer. This may be due to substantial overlap between standardized uptake values (SUVs) from active inflammatory processes with those of malignant lesion.

## INTRODUCTION

Approximately, 70% of transitional cell carcinomas (TCC) of the bladder present as a superficial lesion and nearly 20-25% of newly diagnosed bladder cancers are muscle invasive and 5-10% are metastatic at initial presentation (1). The most frequent site of metastasis is the pelvic lymph nodes. Among the pelvic lymph nodes, the obturator nodes and the external iliac nodes are involved most commonly (74% and 65% of the time respectively). Pre-sacral nodes (25%), common iliac nodes (20%) and para-vesical nodes (16%) are involved with TCC less often. Other distant sites of metastasis of TCC include bone, lung, skin, liver and less commonly the brain and meninges. Few patients with distant metastases survive 5 years. Clearly, the identification of metastases affects the decision-making process for tumor thought to be locally invasive (1).

Radical cystectomy is the standard surgical treatment for muscle invasive urinary bladder cancer. In patients undergoing cystectomy, nodal tumor burden with pathologic evidence of lymph node metastasis is a major prognostic variable (2). Therefore, the accuracy of lymph nodal staging is crucial since treatment options differ significantly according to stage. Lymph node staging is a challenge. With the imaging technologies growing fast 18F-FDG PET has gained popularity because it not only identifies the tumour site but also determines the metabolic activity of metastatic cell and hence determines either regional or distant metastasis. Whereas CT scanning has the limitations that it can only describe the size of the lymph nodes, PET/CT identifies the metastasis in those lymph nodes, which otherwise can be a simple reactive hyperplasia. Therefore, it sounds reasonable to try this technology to improve detection of lymph node metastasis in muscle invasive transitional cell carcinoma of urinary bladder.

The reported accuracy of CT/MRI for nodal staging ranges from 70-90% with false negative rates of 25%-40%. In a study by Stojovska-Jovanovska et al. (3), it was found that primary tumor staging was correct in 55.6% with CT, 56.7% with conventional MRI and in 86.7% with dynamic MRI meaning that none of these modalities are 100% accurate. Therefore, there is a need for alternative functional imaging modality like PET/CT. PET is the newer modality of investigation, which can be used to detect the primary tumor and its metastatic lesion, which have high metabolic activity where FDG is picked up by the hypermetabolic cells (4–7). The current study was designed to determine whether PET/CT can accurately detect nodal metastasis, which were later confirmed by histopathology, the gold standard for nodal metastasis.

## MATERIALS AND METHODS

A total of fifteen patients with muscle invasive TCC of the urinary bladder undergoing radical cystectomy were included during the study period between August 2009 and October 2010. Radical cystectomy was performed by the same surgeon who was blinded to the PET-CT result. Informed consent was obtained from them to take part in the study. Patients with distant metastasis, high blood sugar level (>150mg%), patients who had urinary tract infection, patients who received chemotherapy and radiotherapy, who underwent partial cystectomy or diagnosed to have acute cystitis (CRP>1mg%) were excluded from the study. Patients with positive urine culture were treated with appropriate antibiotics and were included once repeat urine culture had become sterile. History, clinical examination, laboratory tests and cystoscopy findings were recorded. All patients had undergone radical cystectomy with lymph node dissection.

### FDG-PET Imaging

Patients were subjected to whole body PET-CT with dedicated BGO PET-CT scanner, at the department of Nuclear Medicine. All patients were asked to fast for 6-8 hours prior to the scan, blood sugar level was measured for all patients. After confirming acceptable blood sugar level (mean of 110.5mg/dL), 6.5MBq/kg of 18F-FDG was administered. In addition to oral contrast mixed with water, intravenous contrast was also administered via 18G venflon 500mL normal saline was started and 20mg of furosemide was given to all patients 10-15min after FDG injection. This was performed in order to avoid accumulation of FDG metabolite in the urinary bladder causing difficulty in analysis of lymph nodes. Patients were asked to void frequently and were made to wait in the dark room for almost one hour after injection of FDG. Following this, patients were subjected to scanning from head to middle of the thigh. The image reconstruction was done with inbuilt matrix system using reconstruction algorithm.

All patients demonstrated high level of tracer activity in urinary bladder when standard PET/CT images were taken immediately after FDG injection, which obscured the adequate visualization of the tumor. Delayed images were taken after intravenous hydration and furosemide injection to clear the bladder, which reduced the bladder activity to background level.

### PET Data Analysis

The PET images were reviewed on high resolution display monitor in sagital, coronal and transverse sections by physicians of the nuclear medicine department who had an experience of more than five years in this field of nuclear medicine. The observers were blinded to the prior diagnostic work up data. Nodes greater than one centimetre on CT and SUVmax more than 2.5 were considered positive on CT and PET respectively. For assessment of accuracy of staging all imaging data were compared to the gold standard, which was histopathology.

### Specimen Extraction, Labelling And Transport

All patients were operated by the same surgeon. Urinary bladder and regional lymph node specimen were packed in formalin containing packets separately labeled. Histopathological examination was done by the same pathologist who was also blinded to the imaging result.

### Correlation of imaging tests and pathological results

Any patient who had positive pelvic nodes on CT and or PET found to have histologically positive nodes was considered as true positive. Any patient who had positive node on imaging but negative on histopathology was labelled as false positive for nodal staging. Positivity was considered for a given patient with any number of nodes positive on histology. Therefore for final statistical analysis any patient found to have either single node or multiple node positive on histology were considered gold standard positive.

### statistical analysis

The sensitivity, specificity, positive predictive value and negative predictive value were calculated using standard definition.

## RESULTS

Age of our patients ranged from 34 to 67 years, with mean age of 53.4 years. Out of 15 patients, 14 were men (93.3%). Twelve patients (60%) were smokers. None of them was diabetic.

All of them had muscle invasive TCC on histological examination of TURBT specimens. All the patients had undergone open radical cystectomy and standard lymphadenectomy. A total of 150 pelvic lymph nodes from 15 patients were removed out of which 68 nodes were from patients who had positive nodes on PET-CT.

### Analysis of CT and Lymph Nodes Examination

**Figure 1:**
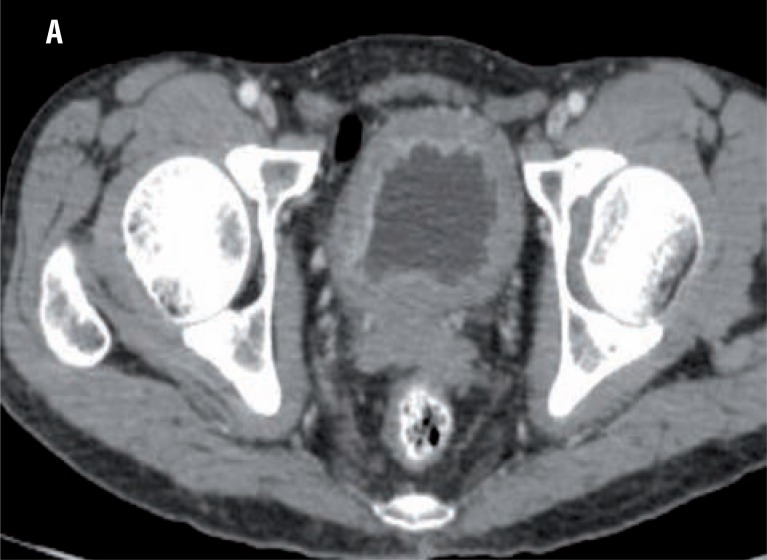
CT showing enhancing soft tissue lesion in the urinary bladder suggesting neoplastic lesion.

In all the 15 patients CT detected the primary lesion as it enhanced after giving contrast because of its vascularity (Figure-1). Five patients showed perivesical stranding or loss of fat planes around the urinary bladder and on final histopathology only one patient showed involvement of the serosa of the bladder and surrounding tissues.

**Figure 2:**
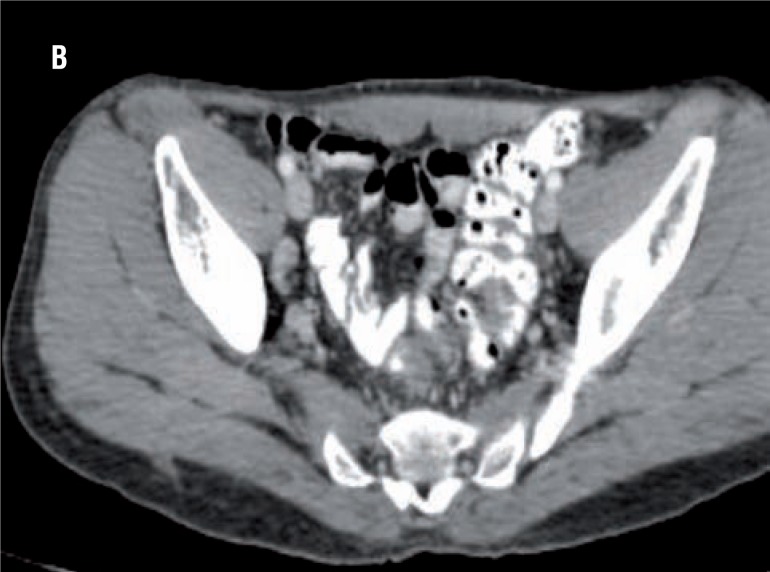
CT showing enlarged lymph node in the pelvis.

**Figure 3:**
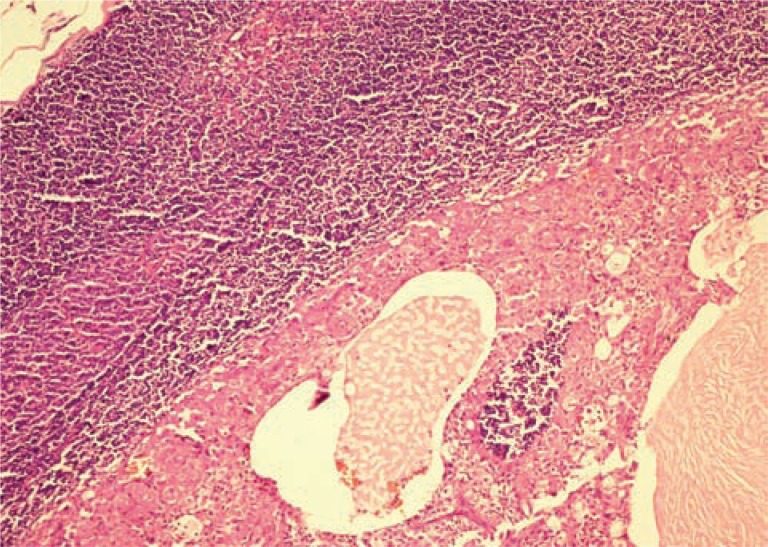
Histopathology showing lymph node metastasis h&E X 20.

Nine patients showed enlarged regional lymph node on CT scan suggestive of metastasis (Figure-2). The result of CT was compared with the gold standard of the lymph nodes obtained from the radical-cystectomy specimen of the same patients. However, only 3 out of the 9 patients actually had metastasis in the lymph nodes as evidenced on histopathology (Figure-3). The other patients (n=6) showed reactive lymph node hyperplasia. Sensitivity, specificity, positive predictive value and negative predictive value of CT alone was 100%, 50%, 33.5% and 100% (Tables 1 and 2).

**Table 1:** CT and PET-CT accuracy for lymph nodes.

		Histopathology of Lymph Nodes		
		Gold standard Positive	Gold standard Negative	
CT	**Positive**	**TP = 3**	**FP = 6**	→ PPV = TP / (TP + FP) = 3 / (3 + 6) =3/9 **= 33.3%**
	**Negative**	**FN = 0**	**TN = 6**	→ NPV = TN / (FN + TN) = 6 / (0+ 6) = 6/6 **100%**
		↓ Sensitivity = TP / (TP + FN) = 3 / (3 + 0) = 3/3 **100%**	↓ Specificity = TN / (FP + TN) = 6/ (6 + 6) = 6/12 **=50%**	
PET-CT	**Positive**	**TP = 3**	**FP = 5**	→ PPV = TP / (TP + FP) = 3 /(3 + 5) = 3/8 **= 37.5%**
	**Negative**	**FN = 0**	**TN = 7**	→ NPV = TN / (FN + N) = 7 / (0+ 7)= 7/7 **100%**
		↓ Sensitivity = TP / (TP + FN) = 3 /(3 + 0)= 3/3 **100%**	↓ Specificity = TN /(FP + TN) = 7 /(5 +7)= 7/12 **= 58.3%**	

**TP** = true positive; **TN** = true negative; **FP** = false positive; **FN** = false negative; **PPV** = positive predictive value; **NPV** = negative predictive value; **PET** = positron emission tomography; **CT** = computed tomography

**Table 2:** Comparison of PET/CT versus CT in terms of nodal detection.

Parameters (%)	PET/CT	CT
Sensitivity	100	100
Specificity	58.3	50.0
PPV	37.5	33.5
NPV	100	100

### Analysis of PET/CT and Lymph Nodes Examination

**Figure 4:**
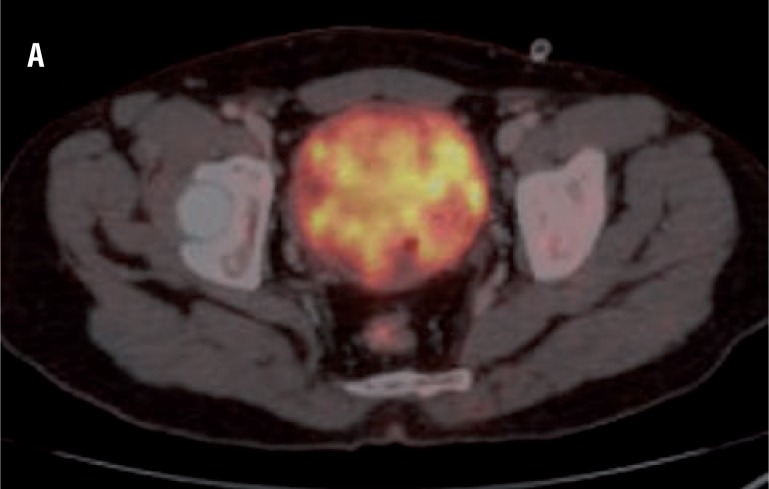
High FDG uptake is seen in soft tissue mass in the bladder suggestive of primary lesion.

In all 15 patients PET/CT detected hyper-metabolic lesion in the urinary bladder suggesting the primary tumour which was clearly visualized after diuresis with furosemide (Figure-4).

**Figure 5:**
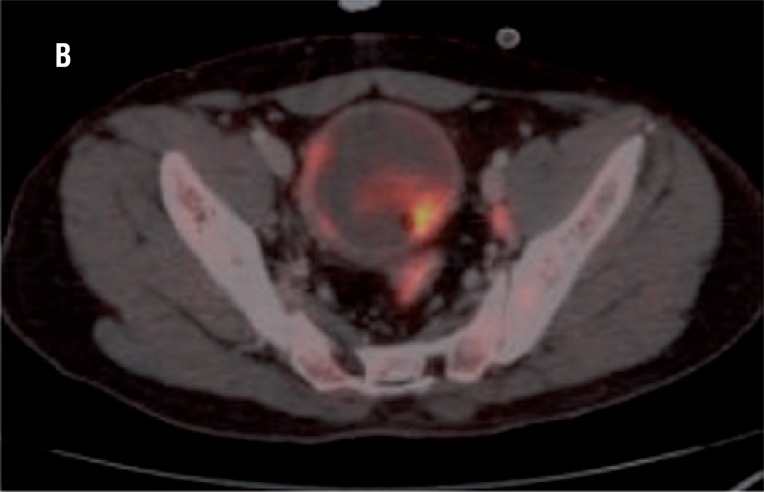
PET / CT showing moderate FDG uptake in left internal iliac nodes (arrow).

Eight patients showed high FDG uptake by the regional lymph nodes suggestive of metastasis. PET along with the CT differentiation was used to find out the site of the positive lymph node. The result of PET/CT was compared with the gold standard, which was histopathology. Among the 8 patients with positive lymph nodes on PET/CT (Figure-5), only 3 had metastasis, and 5 patients showed reactive lymph node hyperplasia. Sensitivity, specificity, positive predictive valve and negative predictive value was 100%, 58.3%, 37.5% and 100%, respectively (Tables 1 and 2).

## DISCUSSION

Muscle invasive urinary bladder tumor is best treated by radical cystectomy which is a high complex procedure. Majority of these patients are elderly with comorbidities and will be subjected to increased risk of procedure related complications. Cystectomy in the presence of nodal disease is controversial. Extent of lymph node dissection and number of nodes retrieved have important impact on outcome in patients with muscle invasive bladder cancer (8–10). During cystectomy more meticulous and extended node dissection improves disease specific survival (11, 12). Therefore, accurate staging of lymph nodes can avoid unnecessary radical cystectomy. However, the fact remains that the most accurate method to stage regional lymph nodes remains regional lymph node dissection. Regional lymph node dissection is usually performed at the time of radical cystectomy at a single operative setting.

CT scan detects only enlarged lymph nodes. Most of the time these are reported on histopathology as reactive lymph node hyperplasia. Therefore, it is not justified to discard the morbid yet curative procedure like radical cystectomy on the basis of traditional CT findings alone. Many of these patients will be under-treated if we deny the gold standard treatment leading to reduced disease specific survival. There is still a significant amount of “mis––staging” (40%) of bladder tumor with MRI. Future advances in MRI, including endorectal coil, enhancement and dynamic contrast imaging may make it more accurate, but they have still not been validated.

The role of PET scanning has also been investigated for use in invasive TCC (Table-3). PET provides images of physiological and metabolic processes. A limitation of PET is the lack of an anatomical reference frame. The combined PET/CT device offers optional fusion of images, which allows the localization of functional findings detected by PET in morphological structures as shown by CT during imaging procedure. FDG, the most commonly used radiopharmaceutical for PET, is unsuitable for evaluation of bladder cancer due to intense accumulation in the urine (13–17). More specific tracers like (11) carbon labeled choline, which are not excreted in the urine fared equally in terms of nodal staging.

**Table 3:** Contemporary series on role of PET/CT for pelvic nodal staging.

Authors	PET Tracer	Number of pts	Sensitivity	Specificity
Drieskens et al. (19)	18F-FDG	55	60	88
Goodfellow H et al. (22)	18F-FDG	233	69	95
Liu et al. (23)	18F-FDG	46	76.9	97.1
Li et al. (24)	18F-FDG	73	75	-
Gofrit et al. (25)	11C-choline	18	100	-
Picchio M et al. (26)	11C-choline	27	62	
Current study	18F-FDG	15	100	58.3

Ahlstrom et al. (17) had compared (18) FDG PET with (11) C-methionine PET and found it suitable for detecting primary tumour but it was not superior. Heicappell et al. (18) reported that in a case series of 8 patients, 3 had metastatic lymph nodes out of which 2 patients showed uptake by PET with a sensitivity of 66.7%.

In our study, the sensitivity was 100% but the specificity was as low as 58.3%. This low specificity is the major concern for its routine use in clinical practice. In the current study CT alone was equally sensitive (100%) as PET/CT. However, it also lacks high specificity in terms of node detection. The only noticeable thing was the negative predictive value which was 100%. That means if a patient has no detectable nodes on CT it will be unlikely to find histologically positive nodes. But the major concern for the physician is for the patients with positive nodes where one has to decide whether to go ahead with complex surgery like radical cystectomy or not. The current study reveals that PET/CT is no better than CT alone for nodal staging. Similar findings had been discussed by Greet Swinnen et al. (13). Their study involved 51 patients, 13 patients had histologically proven nodal metastasis out of which only six patients had demonstrated increased uptake on PET-CT. For the remaining seven patients PET-CT could not diagnose the nodes. The authors related that accuracy, sensitivity, and specificity of FDG-PET/CT for the diagnosis of node positive disease were 84%, 46%, and 97%, respectively. When analysing the results of CT alone, it was observed accuracy of 80%, sensitivity of 46%, and specificity of 92%. The authors found no advantage for combined FDG-PET/CT over CT alone for lymph node staging of invasive bladder cancer or recurrent high-risk superficial disease. In that study the sample size was large and hence more lymph node positive patients were involved, when compared to our study. We had only three patients with metastatic involvement of lymph nodes which was detected by PET/CT hence sensitivity was high.

On the contrary, Drieskens et al. (19) in their series of 55 patients found a sensitivity of 60% and specificity of 88% and they concluded that PET/CT has an advantage over CT in detecting distant lymph nodes. Most investigators feel that the use of FDG-PET has a limited utility for diagnosis and monitoring of urological tumors due to the erratic uptake of the isotope (20, 21). Goodfellow et al. (22) reported that sensitivity and specificity of the CT scans for pelvic LN involvement were 45% and 98%, respectively (N=93).

The small sample size was the main limitation of our study, which can explain the 100% sensitivity observed. Another major limitation of this study was the number of lymph nodes retrieved during surgery. We have dissected an average of 10 lymph nodes per patient. In 8 patients who had PET/CT positive for lymph nodes, 68 nodes were examined on histology, with an average of 8.4 lymph nodes per patient, which is a bit low for accurately identifying all the metastatic lymph nodes as reported by Herr HW et al. (8). These authors concluded that at least 9 lymph nodes should be studied to define nodal status accurately.

In order to overcome the limitations of FDG PET-CT, Li H et al. (24) had used dual-phase (18) F-FDG PET/CT with oral diuretics and achieved the detection rate of 75.0% (6/8) for lymph nodes. Gofrit et al. (25) noticed uptake of (11) C-choline in lymph nodes as small as 5mm (standardized uptake value 3.8±1.4). Picchio M et al. (26) compared CT with (11) C-choline PET amongst 27 patients. Sensitivity for lymph node involvement was 50% by CT and 62% by (11) C-choline PET. The authors concluded that (11) C-choline PET is superior to CT for nodal staging. However, it is obvious that majority of studies confirm that PET-CT with FDG or choline has limited advantage over conventional CT in staging lymph nodes in patients with bladder carcinoma. The current study confirms this point of view.

## CONCLUSIONS

CT detects only enlarged lymph nodes but it tells nothing about the nature of the nodes in terms of presence or absence of metastasis. Theoretically, PET/CT could detect the lymph nodes harbouring metastasis correctly. This study reveals the sensitivity of PET/CT for detection of primary lesion is 100% but in terms of nodal staging its accuracy is low. Therefore, at this stage we cannot say that PET/CT is a better imaging technique compared to CT, to detect malignant lymph nodal involvement. In addition, PET/CT is time consuming, costly and not accessible at every centre. Therefore at the moment we cannot recommend that PET/CT should be an essential tool for the urologist managing invasive bladder cancer.
